# Digital Twin Models in Atrial Fibrillation: Charting the Future of Precision Therapy?

**DOI:** 10.3390/jpm15060256

**Published:** 2025-06-16

**Authors:** Paschalis Karakasis, Antonios P. Antoniadis, Panagiotis Theofilis, Panayotis K. Vlachakis, Nikias Milaras, Dimitrios Patoulias, Theodoros Karamitsos, Nikolaos Fragakis

**Affiliations:** 1 Second Department of Cardiology, Hippokration General Hospital, Medical School, Aristotle University of Thessaloniki, Konstantinoupoleos 49, 54642 Thessaloniki, Greece; aantoniadis@auth.gr (A.P.A.); nfrag@auth.gr (N.F.); 2 First Cardiology Department, School of Medicine, Hippokration General Hospital, National and Kapodistrian University of Athens, 12462 Athens, Greece; panos.theofilis@hotmail.com (P.T.); vlachakispanag@gmail.com (P.K.V.); nikiasmilaras@gmail.com (N.M.); 3 Second Propedeutic Department of Internal Medicine, Faculty of Medicine, School of Health Sciences Aristotle, University of Thessaloniki, 54124 Thessaloniki, Greece; patoulias@auth.gr; 4 First Department of Cardiology, Aristotle University Medical School, AHEPA University General Hospital, 54636 Thessaloniki, Greece; tkaramitsos@auth.gr

**Keywords:** atrial fibrillation, digital twin, left atrium, computational modeling, stroke risk, antiarrhythmic drugs, catheter ablation, precision cardiology, thrombogenicity, personalized medicine

## Abstract

Atrial fibrillation (AF) is the most common sustained arrhythmia and a major contributor to stroke and cardiovascular morbidity. However, current approaches to rhythm control and stroke prevention are often limited by variable treatment responses and population-based risk stratification tools that fail to capture individual disease mechanisms. Digital twin technology—computational models built using patient-specific anatomical and physiological data—has emerged as a promising approach to address these limitations. In the context of AF, left atrial (LA) digital twins integrate structural, electrophysiological, and hemodynamic information to simulate arrhythmia behavior, therapeutic response, and thromboembolic risk with high mechanistic fidelity. Recent applications include stroke risk prediction using computational fluid dynamics, in silico testing of antiarrhythmic drugs, and virtual planning of catheter ablation strategies. These models have shown potential to enhance the personalization of care, offering a more nuanced and predictive framework than conventional scoring systems or imaging alone. Despite promising progress, challenges related to model personalization, computational scalability, and clinical validation remain. Nevertheless, LA digital twins are poised to advance the precision management of AF by bridging in silico modeling with real-world decision-making. This review summarizes the current state and future directions of left atrial digital twin models in AF, focusing on their application in stroke risk prediction, pharmacologic decision-making, and ablation strategy optimization.

## 1. Introduction

Atrial fibrillation (AF) is the most prevalent sustained arrhythmia, affecting over 40 million individuals globally, and is associated with a fivefold increased risk of ischemic stroke, significant morbidity, and substantial healthcare costs [1,2,3,4,5,6,7]. Despite the availability of rhythm- and rate-control strategies, therapeutic outcomes remain suboptimal and highly heterogeneous—reflecting the complex interplay between the electrical, structural, and hemodynamic remodeling of the atria [8,9,10,11,12]. While catheter ablation and antiarrhythmic drugs (AADs) represent the mainstays of rhythm control, both approaches are challenged by limited efficacy, unpredictable responses, and variable recurrence rates [13,14,15,16,17]. Similarly, current approaches to stroke risk prediction, such as the CHA_2_DS_2_-VASc score, rely on clinical surrogates that fail to account for the highly individualized nature of atrial thrombogenesis [18,19,20].

In recent years, the concept of a cardiac digital twins—a patient-specific computational model replicating the structure, electrophysiology, and flow dynamics of the heart—has emerged as a promising paradigm to overcome these limitations [21,22,23,24,25,26]. Enabled by advances in imaging, high-performance computing, and physiological modeling, digital twins of the left atrium (LA) can simulate arrhythmia mechanisms, predict treatment response, and quantify stroke risk with unprecedented mechanistic fidelity [27,28]. Unlike traditional risk models or anatomical imaging, these models integrate anatomical, electrical, and functional data into cohesive in silico environments, allowing for the direct interrogation of therapy efficacy and disease behavior on a per-patient basis.

In the context of AF, digital twin modeling has been applied across three primary domains: (1) stroke risk stratification, using computational fluid dynamics to assess left atrial appendage (LAA) hemodynamics and prothrombotic potential [29]; (2) personalized pharmacologic therapy, including in silico drug testing, gene–drug interaction analysis, and population-based virtual screening [30]; and (3) ablation strategy optimization, where digital simulations of wavefront propagation and rotor dynamics inform lesion design and substrate targeting [31].

This review provides a comprehensive synthesis of recent advancements in LA digital twin modeling, highlighting personalized clinical applications in AF management, mechanistic insights uncovered through virtual experimentation, and the evolving translational landscape.

## 2. Defining Digital Twins in Atrial Fibrillation: Principles and Potential

Digital twins are high-fidelity, computational replicas of physical systems that dynamically integrate patient-specific data to simulate biological behavior under a variety of physiological or therapeutic conditions [32,33,34]. Originally developed in the industrial and engineering domains, the concept has evolved substantially in healthcare, particularly in cardiovascular medicine [32,33,35,36,37,38]. In the context of AF, digital twin models aim to replicate the anatomy, electrophysiology, and hemodynamics of an individual’s atria, providing a powerful platform for mechanism-driven diagnosis, treatment simulation, and risk prediction [39].

A fundamental distinction between digital twins and traditional computational models lies in their level of personalization and dynamic adaptability [40]. While classical models often simulate generalized disease mechanisms using averaged parameters, digital twins are constructed using multimodal patient-specific inputs—such as imaging (CT, MRI), electrocardiography, electroanatomical mapping, and clinical variables—enabling the individualized simulation of arrhythmogenic mechanisms, drug responses, and procedural outcomes [27].

Digital twin frameworks in cardiac electrophysiology may be delineated into three progressive categories [41]:

• Mechanistically related models replicate general electrophysiological behavior through first-principle biophysical simulations, but lack direct calibration to patient-specific data;
• Functionally similar digital twins are constrained by cohort-specific observations and aim to reflect interpatient variability across defined populations, often serving as the basis for in silico trials or population-level prediction;
• Functionally equivalent digital twins represent the highest level of fidelity, being quantitatively calibrated to mirror the structure and function of a single patient’s atria, and thus capable of supporting individual clinical decision-making through predictive simulation.

In AF, the implementation of digital twins has primarily focused on LA remodeling and electrical propagation dynamics, enabling virtual assessments of ablation strategy, antiarrhythmic drug effects, and thromboembolic risk [41]. These models are particularly suited to simulating dynamic and heterogeneous AF substrates, including fibrosis burden, conduction heterogeneity, and restitution properties—factors known to influence therapy response but poorly captured by clinical imaging or scoring systems [41].

The construction of an atrial digital twin typically involves two stages:

1. Anatomical twinning, wherein imaging data (e.g., LGE-MRI or contrast CT) are segmented and meshed to reconstruct the atrial geometry, including structural heterogeneities like fibrosis or scarring.
2. Functional twinning, wherein model parameters such as conduction velocity, action potential duration, and ion channel kinetics are iteratively adjusted to replicate patient-specific electrical behavior, often using ECGs, electrograms, or pacing responses for calibration.

Emerging efforts also emphasize snapshot-based longitudinal twinning, in which digital twins are updated over time using serial data from clinical monitoring or wearable sensors [24,41]. This dynamic updating capability holds particular relevance in AF, where disease progression, rhythm status, and treatment responses evolve over months to years [24,41].

However, despite their potential, the current implementations of digital twins in AF often remain limited to functionally similar models, and most applications rely on fixed calibration snapshots rather than continuous updates [25]. Moreover, real-time use in clinical settings is challenged by the high computational cost of organ-scale simulations, especially those requiring sub-millimeter spatial resolution to resolve reentrant circuits or rotor cores accurately.

Nonetheless, the rapid maturation of simulation platforms, advances in data acquisition (e.g., high-resolution CT, novel MRI sequences), and growing interest in integrating omics and wearable-derived physiology suggest a future in which atrial digital twins become routine tools in precision electrophysiology [42]. By enabling patient-specific, hypothesis-driven simulations, they offer a bridge between mechanistic understanding and individualized care in AF management.

**Figure 1:**
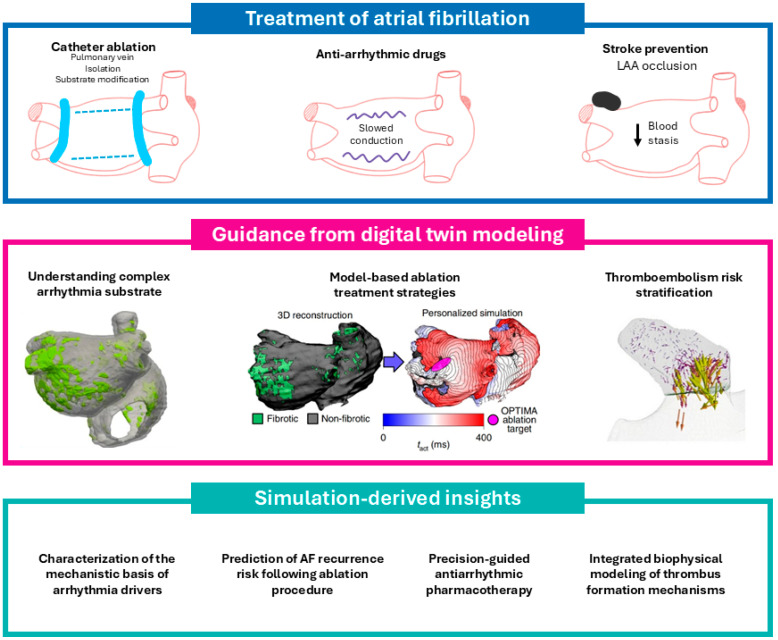
Clinical translation of digital twin modeling in atrial fibrillation. The top panel depicts established clinical strategies for atrial fibrillation (AF) management, encompassing catheter ablation, antiarrhythmic pharmacotherapy, and stroke prevention through anticoagulation. The middle panel illustrates the integrative role of digital twin modeling in augmenting therapeutic decision-making. Specifically, patient-specific models can be employed to delineate the arrhythmogenic substrate, simulate and optimize ablation strategies, and personalize stroke risk stratification. The bottom panel highlights the clinical insights gained through this approach, including mechanistic characterization of AF drivers, refinement of ablation targets, and individualized thromboembolic risk profiling. Collectively, digital twin modeling provides a mechanistic and patient-tailored framework that may enhance precision in AF therapy.

What distinguishes this approach is its ability to model diverse and interrelated clinical dimensions—ranging from hemodynamic stasis in the LA appendage to drug-ion channel interactions and complex reentrant activation patterns. As such, digital twins have found applications across multiple domains of AF management. The following sections examine how this technology has been harnessed to (i) refine stroke risk prediction beyond conventional scoring systems, (ii) guide personalized pharmacologic therapy, and (iii) optimize catheter ablation strategies—collectively positioning digital twins as a foundation for precision electrophysiology. Figure 1 illustrates the evolving role of digital twin modeling in AF management, highlighting its integration with current clinical strategies and its potential to enhance mechanistic understanding, procedural planning, and individualized risk assessment.

## 3. Digital Twin Models for Stroke Risk Prediction in Atrial Fibrillation

Ischemic stroke remains one of the most severe complications of AF, accounting for approximately 15–18% of all cerebrovascular events [43,44,45,46]. While conventional risk stratification tools such as the CHA_2_DS_2_-VASc score are routinely used to guide anticoagulation decisions, they rely on static, population-derived variables and lack the ability to capture individual variations in atrial flow dynamics [47,48,49]. As thrombus formation in AF is intimately tied to LA hemodynamics—especially in the LAA—digital twin models combining patient-specific anatomical and physiological data with computational fluid dynamics (CFD) simulations offer a transformative approach to individualized stroke risk prediction [48].

Recent advancements in CFD-enabled digital twins have enabled the mechanistic quantification of thromboembolic risk by simulating the intra-atrial blood flow and deriving parameters directly linked to Virchow’s triad—such as low velocity, high residence time, and altered wall shear stress [50]. In a proof-of-concept study, Falanga et al. [29] constructed dynamic digital twin model of the LA using contrast-enhanced CT and Doppler data to simulate blood flow in a control subject and an AF patient. Compared to the control, the AF model showed markedly lower flow velocities at the LAA ostium (0.12 vs. 0.28 m/s), higher endothelial cell activation potential (ECAP; 2.23 vs. 1.85 Pa^−1^), and increased residual blood particles (510 vs. 346) after five cardiac cycles, all consistent with a prothrombotic state [29].

Building on this, another investigation expanded their framework to 30 subjects across three groups—controls, paroxysmal AF (PAF), and persistent AF (PsAF) [51]. Using CT-based anatomical models and Doppler-derived inflow conditions, patient-specific CFD simulations were performed under sinus rhythm [51]. The study revealed a stepwise increase in stasis and shear-related indices across AF subtypes: the LAA blood velocity was highest in controls (0.12 m/s) and lowest in PsAF (0.04 m/s), while the ECAP increased substantially from controls (0.93 Pa^−1^) to PsAF (4.77 Pa^−1^). The time-averaged wall shear stress, a surrogate of endothelial shear force, was significantly reduced in AF patients, while the residence time (RRT) increased more than 10-fold in both PAF and PsAF compared to controls [51].

These results underscore how digital twin-driven modeling can uncover thromboembolic risk signatures not captured by CHA_2_DS_2_-VASc. In particular, prolonged RRT and elevated ECAP in the LAA may identify patients with normal clinical risk scores but high biomechanical vulnerability. This offers critical implications for precision anticoagulation and procedural decisions such as LA appendage occlusion (LAAO) [52]. For example, patients with high LAA stasis and impaired washout on digital twin simulations could be considered for LAAO even if their conventional risk score is low, potentially refining the current selection criteria for device-based stroke prevention.

Beyond individual decision-making, these models enable a deeper understanding of how structural and functional remodeling affects LAA flow and thrombogenicity—paving the way for stroke risk assessment that dynamically reflects atrial remodeling, rhythm status, or post-ablation changes [22,53,54]. Despite their promise, current implementations are computationally intensive, and longitudinal validation in prospective trials with clinical endpoints is needed [24].

In summary, digital twin models hold significant promise in enhancing stroke risk prediction in AF by incorporating patient-specific atrial geometry and hemodynamics. As evidence mounts, this approach may serve as a critical adjunct to traditional clinical scores, guiding not only anticoagulation, but also procedural strategies like LAAO and rhythm control in the context of personalized cardiovascular care.

## 4. Digital Twin Models in Pharmacologic Therapy for Atrial Fibrillation: Mechanistic Evaluation and Personalized Prediction

Although catheter ablation has become the primary rhythm control strategy in AF, antiarrhythmic drugs (AADs) remain widely used—either as initial therapy, adjuncts to ablation, or in patients who are not ablation candidates [55,56,57,58]. Yet, the efficacy of AADs is highly inconsistent, reflecting interindividual differences in atrial electrophysiology, substrate remodeling, and underlying genetic predispositions [59,60,61]. This therapeutic variability, combined with risks of proarrhythmia and systemic toxicity, underscores the need for personalized pharmacologic strategies [59,60,61]. In this context, digital twin modeling—realistic, patient-specific computational representations of the atria—offers a powerful platform to simulate, screen, and optimize AAD therapy with unprecedented mechanistic fidelity.

### 4.1. Ion Channel Targeting and Mechanistic Screening in Virtual Atria

Early studies leveraged digital twins to explore how specific ion channel alterations influence AF wave dynamics and inform potential pharmacologic targets. Using tissue models incorporating chronic AF-induced ionic remodeling, Liberos et al. [62] demonstrated that the anti-AF efficacy of L-type calcium current (I CaL) blockade was strongly dependent on the residual sodium and calcium channel function, highlighting the contextual sensitivity of AAD action. Similarly, Scholz et al. [63] incorporated a kinetic model of I Kur inhibition into atrial action potential formulations and showed that its antiarrhythmic effects were contingent on both the state and time dependence of channel blockade.

Further screening studies extended this approach to novel atrial-selective currents. Schmidt et al. [64] computationally modeled the suppression of the TASK-1 (I K,2P) current and found that anti-TASK-1 siRNA prolonged the action potential duration (APD), thereby reducing AF susceptibility. In population-based virtual whole-atria models, Sánchez et al. [65] showed that inhibition of I K1, I NaK, and I Na effectively destabilized reentry by promoting wavefront meandering and reducing dominant frequency (DF). Ni et al. [66] reported that the combined blockade of multiple atrial-predominant K+ currents produced rate-dependent APD prolongation, suggesting synergy in multi-target strategies. These simulation studies established that digital twin platforms can mechanistically dissect drug–target interactions, optimize ion channel selectivity, and predict rhythm outcomes beyond empirical screening.

### 4.2. Genotype-Specific Drug Response: The Case of PITX2 Deficiency

The interplay between atrial genotype and pharmacologic efficacy has been elegantly demonstrated in several digital twin studies focusing on PITX2 haploinsufficiency (PITX2+/−), a well-known AF risk variant. Using computational models integrating clinical imaging, electroanatomical mapping, and ion channel expression profiles, Hwang et al. [67] compared wild-type and PITX2-deficient virtual atria under five AADs (amiodarone, dronedarone, sotalol, flecainide, and propafenone). PITX2-deficient models exhibited shorter APD_90_, lower Smax (maximal APD restitution slope), and reduced DF—all favoring enhanced responsiveness to AADs [67]. Indeed, class IC drugs more effectively prolonged cycle length and reduced phase singularities in PITX2+/− atria (AF termination *p* = 0.018), suggesting genotype-specific responsiveness that is not currently accounted for in guideline-directed therapy [67].

Expanding on this, Jin et al. [68] evaluated the effects of virtual CPVI and virtual AADs (amiodarone, dronedarone, flecainide) in PITX2+/− vs. wild-type atrial models from 25 patients. While CPVI efficacy was genotype-independent, AADs showed significantly higher defragmentation rates in PITX2-deficient models (42% vs. 27%, *p* = 0.014), with accompanying reductions in DF (*p* < 0.001) and Smax (*p* = 0.001) [68]. These findings suggest that digital twin modeling can reveal latent gene–drug interactions that modulate electrophysiologic behavior and therapeutic responses.

### 4.3. Spatial Electrophysiologic Remodeling Under AADs

Beyond scalar measures such as DF or APD, digital twins enable the spatial quantification of wave dynamics at a high resolution. In a study of 25 AF patients, Hwang et al. [69] examined spatial changes in the DF and DF coefficient of variation (DF-COV) across 10 LA segments after the virtual administration of five AADs. DF decreased in a dose-dependent manner (*p* < 0.001), particularly in pulmonary vein (PV) regions compared to extra-PV zones (*p* < 0.001), and episodes of AF defragmentation were characterized by lower DF and higher DF-COV (*p* < 0.001 each), suggesting spatially destabilized but less organized activity under effective drug conditions [69].

This spatial analysis also revealed that regions with high Smax (≥1.4) exhibited lower DFs under AAD exposure, providing a potential mechanistic link between restitution properties and pharmacologic termination [69]. These findings suggest that drug efficacy may not only depend on bulk ionic modulation, but also on the regional susceptibility of the atrial substrate—data that can be uniquely extracted through digital twin analysis [69].

### 4.4. Clinical Translation: The Virtual Amiodarone Test

A landmark demonstration of translational applicability was recently reported through the development of a digital twin-guided “virtual amiodarone test” in a cohort of 115 patients following atrial fibrillation ablation [30] (Table 1). Patient-specific LA models, reconstructed from computed tomography and electroanatomical mapping data, were exposed to simulated escalating doses of amiodarone [30]. Simulations revealed a dose-dependent increase in APD_90_ and a decrease in the peak upstroke velocity (dV/dt) (*p* for trend <0.001), accompanied by progressively higher rates of virtual AF termination at therapeutic drug concentrations [30]. Patients whose digital twins demonstrated the termination of AF at low or high simulated doses were classified as “effective” responders [30]. Clinically, this group exhibited a significantly lower one-year recurrence rate (20.8% vs. 45.1%; adjusted hazard ratio 0.37, *p* = 0.046) compared to patients with ineffective virtual responses [30]. This study offers the first direct correlation between in silico drug response prediction and real-world clinical outcomes, proposing a novel paradigm for individualized antiarrhythmic drug selection.

### 4.5. Toward a Digital Pharmacology Paradigm in AF

As digital twin modeling becomes increasingly refined, its role in AF pharmacotherapy is poised to expand across several domains:

• Drug screening: High-throughput testing of candidate compounds across heterogeneous substrates (e.g., fibrotic vs. non-fibrotic, atrial-selective vs. non-selective) using large in silico populations;
• Genotype-informed therapy: Tailoring AAD selection based on the electrophysiological consequences of common AF risk alleles (e.g., PITX2, SCN5A);
• Toxicity and proarrhythmia prediction: Classifying compounds by proarrhythmic risk profiles using virtual phenotyping (e.g., as shown by Sanchez de la Nava et al. via I_k1_ weighting in random forest models);
• Adaptive therapy planning: Iteratively updating drug models in real time using patient response data and integrating with ablation strategies for hybrid digital twin-guided management.

## 5. Digital Twin-Guided Approaches to AF Ablation: Mechanistic Insight and Clinical Translation

Digital twin models have emerged as a transformative tool for individualized AF ablation planning [24,25,28,41]. These computational frameworks replicate patient-specific LA anatomy, electrophysiological (EP) properties, and arrhythmogenic substrates, enabling simulation-based guidance of ablation targets [24,41]. Multiple studies—ranging from in silico explorations to multicenter randomized trials—have demonstrated their feasibility, mechanistic validity, and clinical utility in PsAF, where empirical ablation strategies often fall short.

### 5.1. Mechanistic Foundations and Driver Mapping

Seminal in silico studies established how digital twins can mechanistically map AF initiation and maintenance. McDowell et al. [71] constructed LGE-MRI-based LA models with patient-specific fibrosis and myofibroblast remodeling, revealing that rotors localized within 378–1052 μm of fibrotic borders—termed “sweet spots”—and ablation of these zones consistently rendered AF non-inducible. Deng et al. [72] tested sensitivity to EP variability and found that modest changes (±10% in APD or CV) resulted in altered rotor anchoring in 20–65% of simulations, emphasizing the importance of incorporating dynamic tissue properties into ablation planning.

Corrado et al. [73] added a machine learning (ML) layer, training support vector machines on >3 million datapoints across 10 patients. They showed that short APD and slow CV accurately predicted PS tethering sites (91% accuracy, rising to 95% with atrial size), suggesting that localized substrate classifiers may enhance driver localization beyond fibrosis mapping alone.

### 5.2. Strategy Testing and Computational Ablation Selection

Several trials evaluated the use of digital twins to optimize lesion sets by simulating competing strategies (Table 2). In the multicenter CUVIA-AF1 trial, Kim et al. [74] randomized 87 PsAF patients to empirical ablation or simulation-guided strategy selection based on five lesion combinations (e.g., CPVI, posterior box isolation [POBI], anterior line [AL], roof line [RL], and CFAE ablation). The strategy yielding AF non-inducibility with the smallest lesion burden was delivered clinically. The modeling-guided group had a significantly lower recurrence at 12 months (14% vs. 41%; HR 0.29; *p* = 0.005), especially in patients with less advanced remodeling.

Shim et al. [77] applied a similar method using CT-based isotropic models and electroanatomical mapping (NavX). Their simulation-guided approach matched empirical outcomes in efficacy, but improved safety by reducing the lesion area and subsequent atrial tachycardias.

### 5.3. Real-Time Implementation and Restitution-Guided Targeting

The CUVIA-AF2 trial [78] implemented real-time digital twin modeling during ablation. Patients underwent CT-based 3D reconstruction, with virtual DF mapping integrated into the clinical workflow. Compared to PVI alone, the V-DF-guided ablation significantly lowered recurrence (HR 0.51; *p* = 0.016), with no increase in procedural time or complications.

A mechanistic post hoc analysis by Park et al. [79] explored why DF targeting was more effective in some patients. Using the restitution slope (Smax) as a functional marker of substrate stability, they found that low-Smax patients (<1) responded well to DF ablation (20% recurrence), while high-Smax patients (≥1) had poor outcomes (67% recurrence). Smax and DF were inversely correlated (r = −0.52; *p* < 0.001), and co-localization maps showed that stable high-frequency sources in low-Smax zones were most responsive to ablation—providing a novel criterion for patient selection.

### 5.4. Iterative Elimination and Personalized Substrate Neutralization

Moving beyond empirical and anatomical target sets, several groups have used iterative simulation to eliminate all arrhythmia-sustaining mechanisms in digital twin models. Boyle et al. [83] developed the OPTIMA framework, constructing bi-atrial finite element models from LGE-MRI and MRA followed by rapid pacing from 40 sites to induce arrhythmias. Identified RDs and macro-reentry paths were virtually ablated until the substrate was rendered non-inducible [83]. The resulting ablation strategy was imported into the CARTO mapping system and delivered to 10 patients with PsAF (60% with prior failed ablation). All remained non-inducible in silico, and the clinical outcomes were favorable [83].

Azzolin et al. [80] extended this approach across 29 bilayer LA models using the PEERP protocol. Their PersonAL strategy identified high dominant frequency (HDF) zones and iteratively applied targeted connection lines [80]. AF was terminated in all models using only 5–6% of the atrial surface—far less than anatomical or substrate-guided strategies—highlighting the efficiency of mechanism-based lesion design [80].

Post-ablation dynamics have also been examined using digital twins. Hakim et al. [82] showed that 75% of models developed emergent RDs after ablation of the original RD site, and 71% of these matched regions predicted only under EP variability conditions. Ali et al. [81] analyzed pre- and post-ablation LGE-MRI from 12 patients and found that recurrence correlated with preserved or emergent RDs in areas of high fibrosis entropy—pointing to the dynamic nature of AF substrates and the need for predictive modeling beyond anatomical scarring.

### 5.5. Artificial Intelligence and Simulation-Efficient Learning

To circumvent the need for handcrafted features or assumptions, Seno et al. [76] developed a deep reinforcement learning framework, training a deep ablation model (DAM) on membrane potential movies from a 2D tissue simulator [76]. Without prior knowledge of arrhythmia mechanisms, the DAM learned to ablate spiral wave cores, achieving a 74.1% AF termination rate using only a 6.5% tissue area—outperforming rotor-guided (8.5%) and random strategies (12.6%) [76].

Roney et al. [75] combined digital twins with machine learning in a population-based simulation stress test. Personalized LA models were subjected to parameter perturbations to simulate AF recurrence post ablation, and recurrence likelihood was predicted using ML classifiers [75]. Their model achieved an AUC of 0.85—significantly outperforming models using clinical variables alone—underscoring the value of combining mechanistic insight with statistical inference [75,87].

### 5.6. Summary and Perspective

Together, these studies represent the maturation of digital twin methodology from a mechanistic tool to a clinical adjunct. The core advantages of this method include:

- Personalized lesion planning: Digital twins consistently outperform empirical strategies by tailoring lesion sets to each patient’s anatomy and substrate;
- Mechanism-targeted ablation: Models identify patient-specific RDs, rotors, or macro-reentrant circuits that may not be visible during clinical mapping;
- Functional phenotyping: Integration of restitution dynamics (e.g., Smax) enhances stratification and target validation;
- Dynamic simulation: Iterative non-inducibility protocols predict residual substrates and emergent arrhythmias, helping to avoid under-treatment or pro-arrhythmia;
- Translational feasibility: Several frameworks (CUVIA-AF, OPTIMA) demonstrate procedural integration without increasing duration or complication rates.

Future directions include embedding digital twins into real-time, closed-loop ablation systems, incorporating deep phenotyping data (e.g., omics, inflammation), and achieving regulatory clearance as clinical decision support tools. As the field shifts toward mechanism-guided therapy, digital twin models may redefine how electrophysiologists approach ablation—no longer guided solely by anatomy, but by individualized substrate dynamics.

## 6. Limitations and Future Directions

Despite significant progress, several limitations hinder the routine clinical adoption of LA digital twin models in AF management [27,88]. First, model calibration remains a major technical bottleneck. Accurately personalizing electrophysiological and structural properties—especially conduction velocity, action potential duration, and fibrotic architecture—requires the integration of heterogeneous and often incomplete datasets [41]. Despite recent advances, non-invasive imaging modalities continue to fall short in resolving the fine-scale arrhythmogenic substrates—such as interstitial fibrosis and micro-reentry circuits—that are central to AF pathophysiology [89]. Moreover, electrical personalization using standard 12-lead ECGs is inherently limited due to their low spatial specificity, and invasive mapping, while informative, introduces procedural burden and variability [28].

Second, the computational demands of high-resolution, biophysically detailed simulations pose practical barriers [90]. Organ-scale models require sub-millimeter spatial resolution and sub-millisecond temporal discretization to resolve wavefront dynamics accurately, resulting in simulations that may take days to compute, even with advanced high-performance computing infrastructure. This restricts their scalability and limits integration into time-sensitive clinical workflows [91]. While simplified hybrid models and machine learning-accelerated surrogates show promise, their interpretability and generalizability remain to be rigorously validated.

Another limitation is the lack of standardized pipelines and cross-platform reproducibility [92]. Many existing digital twin frameworks rely on institution-specific software or undocumented parameters, which impedes validation and broader dissemination [93,94]. The harmonization of model construction workflows, meshing standards, and calibration routines is essential to support multicenter trials and regulatory reviews.

From a clinical perspective, longitudinal validation is urgently needed. Most current studies are cross-sectional and simulate only a single disease state. The ability of digital twins to predict arrhythmia evolution, response to ablation, or stroke risk over time is largely untested. Furthermore, prospective randomized controlled trials comparing digital twin-guided versus empirical treatment strategies remain rare, though ongoing studies such as the OPTIMA trial (NCT04101539) will provide critical evidence [95].

Moreover, existing studies in this domain are often limited by potential biases arising from small sample sizes, heterogeneous modeling protocols, and inherent assumptions in model construction and parameter calibration. These factors underscore the critical need for the rigorous prospective validation and harmonization of modeling standards to ensure clinical applicability.

Furthermore, while current digital twin applications in AF have focused primarily on stroke risk, pharmacologic therapy, and ablation strategy optimization, future developments should aim to extend these models toward supporting comprehensive AF management—including the evaluation of multimorbidity, frailty, and polypharmacy—key components of integrated care frameworks now emphasized in international guidelines [55,57]. To date, no validated digital twin approaches for this broader application exist, underscoring a valuable area for future research and model development.

Emerging initiatives such as the TARGET consortium [96] are pioneering the development of integrated digital twin frameworks that go beyond atrial modeling to encompass the systemic, cerebral, and vascular dimensions of AF-related stroke. Such comprehensive approaches—leveraging AI, in silico trials, and virtual twin technologies—highlight the broader potential of digital twins to personalize stroke prevention and management strategies across the full clinical spectrum of AF.

Looking ahead, the evolution of digital twins in AF will hinge on several key advancements. Multimodal data fusion, integrating imaging (CT, MRI), electroanatomical mapping, genomics, and real-time wearable data, will enhance model fidelity and update responsiveness. The advancement of “functionally equivalent” digital twins—designed to simulate patient-specific responses across various interventions such as pharmacologic therapy, pacing, or ablation—may enhance the capacity for individualized prediction and therapeutic planning. Concurrently, regulatory frameworks will need to adapt to support the evaluation and integration of simulation-based tools and digital biomarkers into clinical practice [97].

Lastly, an additional and critical consideration is the potential for a digital divide to arise with the implementation of digital twin models, as their commercial deployment and computational demands may limit access in resource-constrained settings. To mitigate this risk and promote equitable adoption, future efforts should prioritize the development of open, interoperable platforms, foster public–private collaborations, and advocate for scalable solutions that can be integrated across diverse healthcare systems globally. Moreover, it is essential that these models are developed and validated using diverse, representative cohorts of patients with AF, to avoid introducing biases that could inadvertently exacerbate disparities in care. To date, much of the existing research in this area has been conducted in relatively homogeneous or single-center populations; ensuring diversity and inclusivity in future model training and validation pipelines represents a key imperative for the field.

## 7. Conclusions

LA digital twin models represent an emergent and potentially transformative paradigm in the pursuit of precision therapy for AF. By integrating individualized anatomical, electrophysiological, and hemodynamic parameters, these computational frameworks enable the mechanistic interrogation of arrhythmogenic substrates, thromboembolic propensity, and therapeutic responsiveness. A growing body of literature underscores the feasibility of digital twin-based approaches to inform patient-tailored pharmacologic regimens, optimize catheter ablation strategies, and enhance thromboembolic risk stratification beyond conventional metrics. Despite persistent challenges—most notably the need for harmonized modeling standards, seamless multimodal data assimilation, and rigorous prospective validation—the field is advancing with promising momentum. With continued refinement and clinical integration, digital twin technologies may become an integral component of individualized AF management in the years ahead.

## Figures and Tables

**Table 1:** Digital twin models in pharmacologic therapy for atrial fibrillation: mechanistic evaluation and personalized prediction.

Author, Year	Study Type	Data Sources	Model Features	Primary Application	Key Findings
Liberos et al., 2016 [62]	In silico modeling study	AP recordings from 149 chronic AF patients	Population of 173 atrial tissue models with ionic remodeling variability	Mechanistic evaluation of ICaL, INa, and rotor dynamics	ICaL blockade terminated AF in 30% of models; efficacy modulated by INa availability; rotor destabilization promoted AF extinction
Scholz et al., 2013 [63]	In silico kinetic modeling study	Human CRN atrial model with AF remodeling	2D tissue simulations with state- and voltage-dependent IKur blockade models	Mechanistic assessment of IKur inhibitors	Slow-recovery IKur blockers prolonged refractoriness and terminated rotors; kinetics critical for antiarrhythmic effects
Schmidt et al., 2019 [64]	Preclinical porcine study with in silico support	In vivo AF models, electrophysiology, simulations	Genetic suppression of TASK-1 (IK2P) via AAV9-siRNA; functional EP validation	Targeting TASK-1 for AF prevention	TASK-1 suppression prolonged APD, restored refractoriness, and reduced AF burden by 81.7% with no ventricular toxicity
Sánchez et al., 2017 [65]	In silico modeling study	3D anatomical atrial models with AP population variability	Six heterogeneous models simulating parasympathetic AF remodeling	Evaluating ionic intervention strategies	IK1, INaK, and INa inhibition destabilized rotors and slowed fibrillation; early repolarization prolongation as anti-AF target
Ni et al., 2020 [66]	In silico population modeling study	Human atrial myocytes and tissue strands	Quantitative Systems Pharmacology framework testing IKur, IKCa, and IK2P blockade	Synergistic antiarrhythmic targeting	Combined K+ current block enhanced positive rate-dependent APD prolongation and reentry suppression
Hwang et al., 2024 [30]	Retrospective study with digital twin simulation	CT imaging and electroanatomical mapping	Patient-specific LA models simulating graded amiodarone dosing	Predicting clinical amiodarone efficacy	Virtual AF termination predicted 1-year recurrence (20.8% vs. 45.1%, HR 0.37); APD90 ↑ and dV/dt ↓ dose-dependently
Hwang et al., 2021 [67]	In silico modeling with PITX2 genotyping	CT imaging and electroanatomical mapping	LA models simulating PITX2+/– vs. WT genotypes and virtual AADs	Genotype-specific AAD response prediction	PITX2+/– models showed greater AF termination with class IC drugs (26% vs. 12%, *p* = 0.018); genotype modulated APD, DF, and PS dynamics
Hwang et al., 2021 [69]	In silico spatial analysis study	CT imaging and electroanatomical mapping (n = 25)	Patient-specific LA models simulating AAD effects spatially	Regional AF dynamics under drug therapy	AADs reduced DF especially in PV regions; higher DF heterogeneity associated with successful AF termination; DF inversely related to Smax
Jin et al., 2022 [68]	In silico modeling with clinical imaging and genotyping	CT imaging, bipolar electrograms, PITX2 genotyping	Patient-specific LA models integrating fibrosis and conduction maps; virtual CPVI and AADs	Evaluating genotype-specific efficacy of ablation vs. AADs	AADs more effective in PITX2+/– patients (defragmentation 49.3% vs. 34.7%, *p* = 0.014); V-CPVI efficacy was genotype-independent
Hwang et al., 2023 [70]	Retrospective single-center study with digital twin simulations	Cardiac CT and electroanatomical mapping (EAM) of 232 AF patients post AFCA	Patient-specific digital twins integrating anatomy, histology, and electrophysiology; virtual testing of 5 AADs (amiodarone, sotalol, dronedarone, flecainide, propafenone) at two doses each	Clinical reproducibility and utility of the virtual AAD test (V-AAD)	Patients treated with the most effective V-AAD had lower 1-year AF recurrence (40.9% vs. 54.1%, *p* = 0.046); recurrence trended lower with ≥2 effective drugs (42.4% vs. 59.3%, *p* = 0.056); supports feasibility of V-AAD for post-AFCA drug selection

Abbreviations: AAD—antiarrhythmic drug; AF—atrial fibrillation; AP—action potential; APD—action potential duration; APD20/APD50/APD90—action potential duration at 20%, 50%, 90% repolarization; AT—atrial tachycardia; BB—Bachmann’s bundle; COV-DF—coefficient of variation in dominant frequency; CRN—Courtemanche–Ramirez–Nattel model; CT—computed tomography; CV—conduction velocity; DF—dominant frequency; dV/dt—maximal rate of voltage rise during action potential upstroke; EP—electrophysiology/electrophysiological; HR—hazard ratio; IK1—inward rectifier potassium current; IK2P—two-pore domain potassium channel current; IKur—ultra-rapid delayed rectifier potassium current; IKCa—calcium-activated potassium current; INa—sodium current; INaK—sodium–potassium pump current; LA—left atrium; ML—machine learning; PS—phase singularity; PV—pulmonary vein; Smax—maximal slope of APD restitution curve; TASK-1—TWIK-related acid-sensitive potassium channel-1; V-CPVI—virtual circumferential pulmonary vein isolation; and WT—wild-type.

**Table 2:** Key studies employing left atrial digital twin modeling for atrial fibrillation ablation planning, personalization, and outcome prediction.

Author, Year	Study Type	Data Sources	Model Features	Primary Application	Key Findings
Sakata et al., 2024 [31]	Prospective clinical study with digital twin modeling	LGE-MRI, electroanatomical mapping	Bi-atrial digital twins with fibrosis and rotor inducibility testing	Mechanism-based ablation targeting	Lesion-minimizing strategy reduced targets by 34% and mitigated AT risk
Roney et al., 2022 [75]	Retrospective modeling with machine learning	LGE-MRI, ECG follow-up	Patient-specific LA models with fibrosis, fiber orientation; stress tests; ML integration	AF recurrence prediction	Combined simulations and clinical data improved AF recurrence prediction (AUC 0.85)
Seno et al., 2021 [76]	In silico study with deep reinforcement learning	2D cardiac tissue simulation	DAM trained on membrane potential movies to generate ablation patterns	Learning ablation strategies	DAM achieved 74.1% AF termination with minimal ablation compared to random or rotor-based strategies
Shim et al., 2017 [77]	Multicenter RCT with virtual modeling	CT + NavX mapping	Isotropic LA models tested with five lesion strategies	Strategy selection in PsAF	Simulation-guided ablation was feasible and not inferior; improved outcomes vs. empirical sets
Baek et al., 2021 [78]	Multicenter RCT with real-time modeling (CUVIA-AF2)	CT + electroanatomical mapping	LA models with fibrosis, fiber orientation, DF analysis	DF-guided ablation during procedure	V-DF ablation lowered recurrence (HR 0.51, *p* = 0.016); completed in standard procedure time
Kim et al., 2019 [74]	Multicenter RCT with simulation (CUVIA-AF1)	CT-based 3D LA models	Monolayer models tested with five lesion sets for optimal choice	Guided lesion selection	Model-based strategy reduced recurrence (HR 0.29, *p* = 0.005); more effective in less remodeled LA
Park et al., 2022 [79]	Post hoc modeling study (CUVIA-AF2)	CT + mapping data	LA models with APD restitution (Smax) and DF integration	Assessing Smax-dependent ablation efficacy	DF ablation effective mainly in low-Smax patients; RD locations inversely related to Smax
Azzolin et al., 2023 [80]	In silico study with clinical mapping	LGE-MRI, electroanatomical mapping	Bilayer models; iterative PEERP induction; 13 ablation strategies tested	PersonAL ablation optimization	Iterative HDF targeting terminated AF in all models using 5–6% of atrial tissue
Corrado et al., 2021 [73]	In silico study with ML classifier	EAM-based LA models	10 LA models with fitted CV/APD; ML prediction of PS tethering	Identifying reentry sites	Slow CV and short APD predicted PS tethering with 91–95% accuracy
Ali et al., 2019 [81]	Retrospective pre-post LGE-MRI study	Pre- and post-ablation LGE-MRI	3D LA models with fibrosis; RD tracking pre/post ablation	Understanding ablation failure	Emergent RDs overlapped fibrosis entropy zones; explained AF recurrence
McDowell et al., 2015 [71]	Proof-of-concept in silico study	LGE-MRI-based LA models	3D models with fibrosis, myofibroblasts, and rotor dynamics	Predicting RD zones	RDs anchored at fibrosis border zones; targeting them rendered models non-inducible
Deng et al., 2017 [72]	In silico sensitivity analysis	LGE-MRI-derived LA models	EP variability tested (±10% APD/CV); RD anchoring analyzed	Assessing EP sensitivity	20–65% of RDs changed locations under varied EP; underlined need for robust modeling
Hakim et al., 2018 [82]	In silico post-ablation RD dynamics study	LGE-MRI-derived models	EPavg models with virtual RD ablation and pacing	Characterizing emergent RDs	Emergent RDs appeared post ablation in 75%; iterative targeting recommended
Boyle et al., 2019 [83]	Prospective feasibility study (OPTIMA)	LGE-MRI + MRA	Bi-atrial finite element models; RD/macro-AT elimination; targets imported into CARTO	Personalized non-inducibility via pre-planned ablation	OPTIMA-guided ablation eliminated inducibility in all PsAF cases; strong clinical feasibility
Lim et al., 2020 [84]	In silico modeling with clinical validation	CT imaging and electroanatomical mapping	3D biatrial models integrating realistic anatomy, voltage maps, fiber orientation, fibrosis, and interatrial connections (BB, posterior/anterior septum, CTI)	Testing the impact of sequential interatrial conduction ablation post-CPVI	Virtual CTI ablation improved AF termination (80% vs. 30%, *p* < 0.001); in clinical cohort (n = 846), CTI ablation reduced 2-year recurrence (HR 0.60, *p* < 0.001)
Boyle et al., 2018 [85]	Retrospective comparative modeling and clinical mapping study	LGE-MRI, ECGI, CT imaging, electrograms in 12 PsAF patients	3D patient-specific bi-atrial models from LGE-MRI; fibrosis distribution; Courtemanche-based membrane kinetics; 30 pacing sites; RD tracking algorithms	Comparison of RD locations predicted by modeling vs. ECGI	RDsim were found in 28 regions vs. 42 for RDECGI; modest spatial agreement (κ = 0.11); 19 regions had both RD types (ECGI+/Sim+); ECGI-driven ablation had higher success when targets overlapped RDsim sites (57% vs. 41%); simulations revealed latent fibrosis-mediated RD sites missed by ECGI; combined simulation–ECGI strategy proposed to improve ablation outcomes
Dasí et al., 2024 [86]	Large-scale in silico trial	Human CT, MRI, EAM data for anatomical/structural calibration; ionic current and ECG calibration	800 virtual AF patients with heterogeneity in atrial anatomy, ionic currents (40 profiles), and low-voltage areas (LVAs); personalized 3D bi-atrial simulations with >7000 treatments tested	Patient stratification for optimal second-line AF therapy post-PVI (ablation and AADs)	Stratification based on LVA presence, atrial size, and ERP guided selection of PWI, MiLine, CTI, Marshall-PLAN, and AADs. LVA ablation in both atria + CTI block yielded 100% efficacy in LVA+ patients; pharmacologic success varied by INa/ICaL density (e.g., amiodarone success 57%). Decision algorithm proposed for individualized therapy based on electrophysiological and structural metrics

Abbreviations: AF—atrial fibrillation; APD—action potential duration; AT/Afl—atrial tachycardia/atrial flutter; CARTO—electroanatomic mapping system (Biosense Webster); CFAE—complex fractionated atrial electrograms; CPVI—circumferential pulmonary vein isolation; CT—computed tomography; CV—conduction velocity; DAM—deep neural network-based ablation model; DF—dominant frequency; EAM—electroanatomical mapping; ECGI—electrocardiographic imaging; EP—electrophysiology/electrophysiological; FEM—finite element model; HDF—high dominant frequency; LA—left atrium; LAA—left atrial appendage; LGE-MRI—late gadolinium enhancement magnetic resonance imaging; LLI—lower lateral isthmus line; LPV—left pulmonary veins; ML—machine learning; MRA—magnetic resonance angiography; PVI—pulmonary vein isolation; PsAF—persistent atrial fibrillation; RA—right atrium; RD—reentrant driver; RDsim—reentrant driver predicted by simulation; RDECGI—reentrant driver mapped by ECGI; RL—roof line; and Smax—maximal slope of APD restitution curve.

## Data Availability

All data generated in this research is included within the article.
